# Exploring the state of evidence on aging with HIV in long-term care: A scoping review protocol

**DOI:** 10.1371/journal.pone.0335131

**Published:** 2025-10-29

**Authors:** Kristina M. Kokorelias, Soo Chan Carusone, Christine Sheppard, Andrew D. Eaton, Cynthia Chui, Dean Valentine, Michael Kirk, Luxey Sirisegaram

**Affiliations:** 1 Section of Geriatric Medicine, Department of Medicine, Sinai Health System and University Health Network, Toronto, Ontario, Canada; 2 Department of Occupational Science & Occupational Therapy, Temerty Faculty of Medicine, University of Toronto, Toronto, Ontario, Canada; 3 National Institute on Ageing, Toronto Metropolitan University, Toronto, Ontario, Canada; 4 Rehabilitation Sciences Institute, Temerty Faculty of Medicine, University of Toronto, Toronto, Ontario, Canada; 5 Department of Health Research Methods, Evidence, & Impact, McMaster University, Hamilton, Ontario, Canada; 6 Factor-Inwentash Faculty of Social Work, University of Toronto, Toronto, Ontario, Canada; 7 Jane Addams College of Social Work, University of Illinois, Chicago, Illinois, United States of America; 8 Department of Medicine, The Temerty Faculty of Medicine, University of Toronto, Toronto, Ontario, Canada; University of New Brunswick, CANADA

## Abstract

**Background:**

The growing population of older adults living with HIV presents unique challenges for long-term care facilities, which are increasingly tasked with supporting residents who require both HIV-specific and geriatric care. Despite advances in HIV treatment that have extended life expectancy, the needs of these individuals in long-term care remain underexplored, and the field lacks a consolidated understanding of how facilities are currently equipped to manage these complexities. This scoping review protocol outlines the approach for synthesizing existing evidence on the experiences, challenges, and care outcomes of aging with HIV in long-term care settings.

**Objective:**

To examine the state of evidence on older adults with HIV in long-term care, providing an overview of current knowledge on the health, social, and systemic factors influencing their care and identifying gaps that may guide future research and practice.

**Methods:**

The team includes knowledge users, including experts by experience, to ensure the findings are grounded in lived realities and practical applicability. Using a scoping review framework by the Joanna Briggs Institute, we will conduct a comprehensive search of literature from inception in the following electronic databases: MEDLINE (R) ALL (Ovid), Embase Classic + Embase (Ovid), Cochrane Central Register of Controlled Trials (Ovid), CINAHL Ultimate (EBSCO), PsycINFO (Ovid), AgeLine (EBSCO), and Scopus to capture studies that address aging with HIV in long-term care settings. Eligible studies will be screened and selected based on criteria focused on relevance to the intersection of aging, HIV, and long-term care. Articles will be screened by two reviewers. Data will be charted and synthesized thematically, allowing for an organized summary of findings on key topics such as physical and mental health, care provision, and facility preparedness.

**Discussion and implications:**

This review will offer an overview of the current state of knowledge on aging with HIV in long-term care facilities, highlighting what is known about care practices, health outcomes, and systemic challenges in these settings. Findings will clarify the breadth and depth of existing evidence and reveal areas requiring further research, thereby informing policy and enhancing care strategies for this population.

## Introduction

The intersection of aging and human immunodeficiency virus (HIV) care is an emerging and complex field, reflecting significant demographic shifts among individuals living with HIV [1–6]. Due to the success of antiretroviral therapy, many people with HIV are now living longer and aging into a phase of life that introduces age-related health challenges alongside ongoing HIV management [7]. The global proportion of people living with HIV aged 50 and older rose significantly, doubling from 8% in 2000 to 16% in 2016, and the number of persons aging with HIV is only expected to grow [8]. In the USA, around 50% of the 1.3 million individuals living with HIV/AIDS (PLWHA) are aged 50 and older, and this proportion is expected to rise to 70% by 2030 [9]. As this population grows, long-term care (LTC) facilities—responsible for providing extensive and sustained care to older adults living with HIV—are increasingly expected to address the specific needs of aging individuals living with HIV [10]. This demographic shift raises critical questions regarding how well LTC facilities are prepared to provide appropriate, inclusive, and effective care for this unique group.

Aging with HIV entails a combination of medical, psychological, and social complexities [11,12]. Individuals who have lived with HIV, particularly for extended periods of time, face accelerated aging [13] and a higher prevalence of comorbidities, such as cardiovascular disease, bone density loss, neurocognitive decline, and certain cancers, which compound typical aging challenges [14,15]. Research suggests that HIV itself, along with long-term antiretroviral therapy use, may contribute to an increased risk of frailty and functional impairments [16]. For aging individuals with HIV, these factors may lead to an earlier and more intense reliance on supportive care services.

The intersection of HIV and aging also introduces unique psychosocial needs, such as addressing HIV stigma and managing mental health conditions [17,18]. Many individuals living with HIV may have a fear of entering long-term care settings due to stigma, past discrimination, or concerns about inadequate healthcare tailored to their specific health needs [19–22], particularly in managing HIV alongside other age-related conditions. Conversely, some express a desire to avoid long-term care due to perceived loss of independence or a lack of culturally competent care [22–24]. Furthermore, many older adults living with HIV experience intersecting stigmas related to aging, sexual orientation, and substance use histories [25–28], which can compound feelings of exclusion and impact their mental health and well-being in LTC settings. These concerns are often compounded by anxieties over the quality of care [24,29], prevalent misconceptions about HIV transmission [30]. The ability of healthcare providers to adequately manage HIV treatment in older age [31], and the potential for social isolation within such settings [32]. Understanding these perspectives is key to shaping policies and practices that ensure better integration of older adults living with HIV into long-term care systems. Moreover, individuals living with HIV are more likely to have strained or limited family relationships, often due to factors such as social stigma, estrangement, or isolation [33]. This lack of a strong support network can increase their need for long-term care services, as they may have fewer resources to rely on for assistance with daily activities, healthcare management, and emotional support.

Despite the growing demand, the capacity of LTC facilities to meet the needs of older adults living with HIV remains inadequately explored [12]. A major challenge in preparing LTC facilities for the influx of older adults with HIV lies in the existing knowledge gap on best practices for their care. Additionally, while the majority of research focuses on the clinical aspects of HIV care, there is an emerging recognition that social and psychological support play an equally critical role in supporting healthy aging with HIV [34]. Historically, LTC models and staff may not be appropriate to meet the complex, multidimensional health and social care needs associated with HIV [11,35–38]. Additionally, while very few LTC facilities have developed specialty care programs [39,40] and many are seeing increasing numbers of residents living with HIV in LTC facilities, evidence on the effectiveness, challenges, and accessibility of these programs for older adults living with HIV remains sparse [41]. Rather, much of the available health service research is concentrated in acute or outpatient settings [42–44], with limited exploration of the long-term and residential care landscape [45].

Consequently, the state of evidence on aging with HIV in LTC settings remains fragmented, with most studies addressing either clinical health needs or the broader social challenges with aging, rather than exploring how these elements intersect to shape lived experiences. Additionally, while some studies have explored attitudes and training needs among LTC staff, little is known about systemic or organizational factors that facilitate or hinder the provision of HIV-informed care in LTC settings [20,46]. Given these knowledge gaps, a comprehensive scoping review is essential to map the existing literature and to delineate what is known and what remains unexplored regarding the intersection of HIV and aging within LTC. This review will consolidate current evidence on the existing models of HIV care, experiences, challenges, and outcomes of older adults living with HIV in LTC facilities, identifying gaps in research, policy, and practice. By doing so, it will provide a foundation for future studies aimed at optimizing care models and developing targeted interventions, ultimately supporting the provision of inclusive, high-quality care for older adults living with HIV in LTC settings. This scoping review will also offer insights that could inform policy makers, healthcare providers, and educators about essential competencies and systemic adjustments needed to better support this population.

Our review will answer, “what is known about older adults living with HIV within long-term care (LTC) settings?”. Our secondary questions are:

1) What is known about the experiences, challenges, and health outcomes of older adults living with HIV residing in LTC facilities? What are potential regional variations in these experiences?
2) What existing models of HIV care are being implemented in LTC settings for living older adults?

In the context of this study, long-term care settings refer to residential settings that provide ongoing care, medical support, and assistance with daily activities to individuals, often older adults, who have chronic health needs or functional limitations [47–49]. These facilities are known by various terms, including nursing homes, assisted living facilities, skilled nursing facilities, and elder care homes, reflecting the range of services and levels of care provided across different contexts [49].

## Materials and methods

### Design

A systematic patient-oriented approach to a scoping review [50] will occur in early 2025. The Joanna Briggs Institute methodology for scoping reviews [51], which is suitable for the broad objective of examining the current state of evidence on aging with HIV in LTC facility settings, will guide the conduct of this review. State of the evidence reviews typically emphasize recently published research to assess current matters and developments in the field [52]. These reviews often highlight new ideas, emerging trends, or gaps in research that have been identified in recent studies [52]. A scoping review was identified to review the state of current knowledge, identify key concepts, and highlight gaps in the literature, thereby providing a comprehensive overview of emerging trends and unresolved issues.

By following scoping review guidelines, particularly those from the Joanna Briggs Institute, this review will systematically capture and synthesize studies, ensuring that the findings accurately reflect contemporary developments and research priorities in aging with HIV within long-term care settings. As such, we will also adhere to best practice guidance for developing scoping review protocols (PRISMA-P) [53] (S1 File), as well as the PRISMA-ScR (Preferred Reporting Items for Systematic Reviews and Meta-Analyses extension for Scoping Reviews) guidelines [54] (S2 File), to ensure comprehensive and transparent reporting. The protocol has also been registered with the Open Science Framework (osf.io/8pmrf).

### Inclusion and exclusion criteria

The forthcoming study will employ a comprehensive search strategy developed in collaboration with a medical information specialist and the research team. The research team will also consult experts from both the HIV and long-term care fields (such as the Canadian HIV/AIDS Clinical Trials Network) to refine our approach and identify key studies. The following databases will be searched: MEDLINE (R) ALL (Ovid), Embase Classic + Embase (Ovid), Cochrane Central Register of Controlled Trials (Ovid), CINAHL Ultimate (EBSCO), PsycInfo (Ovid), AgeLine (EBSCO), and Scopus. The initial search strategy was developed in MEDLINE and tested using a set of target articles provided by the team. The search strategy included subject headings (e.g., Medical Subject Headings, MeSH) and text words for 2 search concepts: 1) HIV and 2) Long-Term Care. Some keywords included: HIV, AIDS, long-term care, residential home, nursing home, and institution. A sample of 200 articles were then screened by the team to identify additional search terms and to improve the search. The draft Medline search was then peer reviewed using the Peer Review of Electronic Search Strategies (PRESS) checklist by another information specialist, who was not involved in search development, to ensure its comprehensiveness and accuracy (S3 File) [56]. The search strategy was then revised with recommendations from the peer review process. The finalized Medline search strategy will be translated to the other electronic databases. The references obtained from these searches will be exported to a reference management system (EndNote), where any duplicate entries will be eliminated [57] before importing into Covidence [58].

### Search strategy and information sources

Reference lists of all included documents will be reviewed.

Any non-English literature found in the searches will be translated using Google Translate for the purposes of analysis [59].

### Search process

Evidence retrieved through the prepared search strategy will be imported into Covidence to allow for systematic screening [58]. The screening process will commence with a pilot test involving the first 50 titles and abstracts to ensure that the reviewers clearly understand the inclusion criteria. Further training sessions and pilot screenings will be conducted until the reviewers achieve an agreement rate of 80% or higher on inclusion decisions. Once the pilot is successful, each title and abstract will be independently screened by two reviewers. The full-text versions of articles assessed as relevant during the title and abstract screening will then be retrieved for further evaluation. Before moving on to the full-text screening, the reviewers will conduct another pilot review of at least 5 full-text documents to confirm that they can reach a consensus of at least 80% agreement on the subset of documents. Upon achieving this level of agreement, each full-text document will be independently reviewed by two reviewers who will decide on its inclusion or exclusion based off **Table 1**.

**Table 1:** outlines the eligibility and exclusion criteria were developed to ensure a comprehensive yet focused selection of studies relevant to the intersection of aging, HIV, and LTC settings.

Criteria	Inclusion	Exclusion	Justification
**Population**	Older adults (aged 50+) living with HIV If a broader population is included, the study must feature subgroup analysis for older adults living with HIV.	Studies that do not specify an age group, focus on individuals under 50, or exclude people living with HIV.	A focus on older adults living with HIV (50+) is essential to explore the unique aging-related health challenges and needs of this population with HIV in LTC settings. In the context of HIV, older age is often defined as aged 50 years and older [55].
**Concept**	Focus on models of HIV care in LTC, health outcomes, experiences, barriers in LTC, psychosocial and physical needs, or staff attitudes towards HIV care	Studies not involving older adults with HIV as a topic in their analysis or studies on other elements in LTC such as medication reviews, that are not directly linked to HIV.	Concentrating on these specific topics will highlight existing care practices, patient and provider experiences, and gaps in HIV care provision in LTC. Limiting to LTC-relevant contexts ensures findings are directly applicable to residential care settings.
**Location**	Research with findings related to LTC settings, including nursing homes and residential care settings (e.g., skilled nursing facilities, long-term care facilities, assisted living facilities, elder care facilities, adult care homes, convalescent homes, supportive housing for older adults, rest homes, personal care homes, geriatric care facilities, intermediate care facilities, board and care homes, senior living communities, aged care facilities)	Studies on HIV and aging with no connection to LTC or residential care settings, including hospitals, rehabilitation centers, retirement homes (that do not provide nursing care), supportive housing, etc. Studies focused only on community-dwelling older adults or those in hospital-based acute care without relevant LTC context	Limiting the context to LTC environments is necessary, as community or hospital-based care differs in resources, care needs, and settings, and thus may not address the unique challenges in LTC facilities.
**Study Types**	Includes primary research (qualitative, quantitative, mixed methods), and case studies.	Opinion pieces, editorials, conference abstracts without full studies or models of care, or publications lacking methodological detail or detail on models of care. Excluding literature reviews, reports, guidelines, policy documents, best practice recommendations,	Including diverse research will help capture a comprehensive view of available evidence. Excluding non-research pieces (e.g., grey literature), non peer-reviewed studies (e.g., theses) and low-detail publications ensures that only high-quality data and recommendations are considered.

In cases where there is disagreement between reviewers regarding the inclusion of a source, this will be resolved through discussion between the reviewers with the involvement of the senior responsible reviewer (LS). The results from the search strategy and screening exercises will be summarized and illustrated in a PRISMA flow diagram, which will provide a clear overview of the study selection process, including the rationale for exclusion of articles at the full-text review stage [54].

### Data extraction

Two independent reviewers will extract data from the identified articles using a data abstraction tool specifically designed for this review. The initial extraction tool will be informed by our knowledge user team, including individuals living with HIV and clinicians/ experts in long-term care, ensuring that it reflects a comprehensive understanding of the relevant issues and perspectives. The information extracted will include key study characteristics, including the title, authors, publication year, country of origin, methodology, research objectives, health delivery issues addressed, study population, identification of any challenges or successes in including people living with HIV, whether individuals with lived experience were involved in the research process (e.g., as co-researchers, advisors, or participants), type of methods used to incorporate the perspectives of people living with HIV/AIDS (e.g., participatory action research, focus groups, interviews) and setting description. Charting will be an iterative process, allowing for modifications to the data abstraction table as the reviewers gain insights from the data, ensuring that all research questions are adequately addressed. For instance, if new categories emerge as relevant to our research questions, they will be incorporated into the table.

Following established methodology for scoping reviews, we will initiate the charting process with a pilot test on 2 articles to evaluate consistency between reviewers and confirm alignment with the scoping review’s objectives. Should any discrepancies arise, the research team will engage in discussions to review and revise the data abstraction template accordingly.

### Data analysis

We will analyze and present the data in a tabular format, including a descriptive numerical summary of the study characteristics alongside a narrative thematic analysis. The thematic analysis will be guided by Braun and Clarke’s approach, which involves several key steps. Initially, we will apply codes to the data that capture the essence of the content [60,61]. These codes will then be organized into potential themes based on observed patterns among similar codes. Our team will review these themes across the entire dataset to ensure they are representative, refining their names for clarity and accuracy. The aspects of the data most suitable for thematic analysis will include theoretical perspectives, the environments under assessment, relevant sectors, scanning modes, and identified limitations.

We will group included studies according to the World Health Organization’s regional classification (i.e., African Region, Region of the Americas, South-East Asia Region, European Region, Eastern Mediterranean Region, and Western Pacific Region). Where sufficient data are available, we will conduct a sub-analysis to compare themes, practices, and policy implications across these regions. This will allow us to explore contextual differences in LTC experiences among older PLHIV and identify region-specific challenges and innovations.

### Patient and public consultation

The impetus for this project was informed by direct conversations with older adults living with HIV, as well as priorities discussed in an advisory group focused on aging and HIV [62]. By including individuals with lived experience as older adults living with HIV, as well as clinicians who care for this population, we have ensured that the research is rooted in real-world concerns and perspectives.

In alignment with best practices for inclusive research, [63] we have included knowledge users with lived experience as an older adult living with HIV, as well as individuals caring for older adults living with HIV (clinicians) in the planning of this review, who will be engaged in the project throughout the conduct and dissemination of the final study. These knowledge users are involved in an advisory committee formed to explore aging and HIV [62]. They will participate in regular meetings to discuss the review’s progress, contribute to decision-making processes, and provide feedback on interim findings. Their involvement will help to ensure that the research design, methodology, and dissemination strategies are attuned to the practical challenges and opportunities within the healthcare landscape. Moreover, during the dissemination of the final study, knowledge users will play a critical role in translating the research findings into actionable recommendations. They will assist in creating tailored communication strategies to reach broader audiences, including stakeholders and practitioners who can implement the findings in their respective fields.

## Results and discussion

The anticipated results of this review are expected to provide a comprehensive synthesis of the state of knowledge on older adults living with HIV in LTC settings. We anticipate identifying key themes related to the experiences of older adults living with HIV and staff, existing models of HIV-specific care within LTC, and the integration of HIV-specific interventions within long-term care frameworks. Additionally, the review aims to highlight gaps in the current evidence base, particularly concerning the unique needs of older adults living with HIV in long-term care, and to outline best practices that can inform future healthcare delivery models. By mapping the current landscape of research and practice, we hope to generate actionable insights that can influence policy decisions, enhance service delivery, and ultimately improve health outcomes for this growing population. A major goal of this review is to expose gaps in the existing literature and evidence base, particularly regarding the unmet needs of older adults living with HIV who reside in LTC settings. These gaps are particularly important to address, as the healthcare needs of this group are often overlooked in mainstream geriatric-HIV care models [45,64]. The identification of these gaps will not only guide future research priorities but will also provide critical information for policymakers and healthcare providers seeking to improve the delivery of care to this group.

The review aims to synthesize a broad range of literature on the experiences, challenges, and outcomes of older adults living with HIV in long-term care settings, providing a holistic understanding of this complex intersection. By mapping the existing evidence, the review will identify critical gaps in knowledge and practice, guiding future research and informing policy development to better address the needs of this population. The review acknowledges the heterogeneity of older adults living with HIV, emphasizing the importance of capturing diverse experiences and needs, which can lead to more tailored and effective care models.

As the HIV population ages, it is essential to understand how the aging process intersects with the unique needs of individuals living with HIV. Older adults living with HIV face distinct health challenges, such as multimorbidity and age-related conditions, which require specialized care strategies in long-term care settings. This review addresses the urgent need to better understand this demographic shift. Long-term care facilities are often ill-equipped to address the specific health and social needs of older adults living with HIV. By identifying knowledge gaps, the review provides evidence that can guide improvements in care, ensuring these facilities are prepared to meet the evolving needs of this population. As public health policies evolve to accommodate an aging HIV population, this review will inform the development of policies and care models that are inclusive and responsive to the needs of older adults living with HIV. By synthesizing current evidence, the review offers practical recommendations that can be implemented at the organizational and policy levels.

### Limitations

The review may still be prone to publication bias, as studies with positive or novel findings are more likely to be published [65]. This could lead to an overrepresentation of certain themes or interventions that are more readily reported or studied. Non-English language studies may still be underrepresented in the search process due to potential limitations in database search capabilities, or challenges in accessing and reviewing studies in other languages. Lastly, the review might not fully account for cultural or regional differences in how HIV care is provided in LTC settings, which could impact the applicability of the findings to specific locations or populations.

First, LTC settings vary significantly across the globe in terms of availability, structure, and regulation [66]. In many countries, institutionalized long-term care is not the predominant mode of supporting older adults [67]. Instead, care is often provided within intergenerational households or through informal community networks. This cultural variability introduces a significant bias toward countries that have established LTC systems, particularly those that are regulated and well-documented enough to be included in academic studies. As a result, the findings and conclusions drawn from this review may not be fully generalizable to settings where formal LTC facilities are either rare or non-existent. This focus may exclude valuable perspectives and practices from middle- and low-income countries, where innovative or informal models of elder care might yield unique insights into fostering engagement in different caregiving contexts.

## Conclusions

The forthcoming state of the evidence scoping review aims to illuminate the current state of evidence regarding older adults living with HIV in LTC settings, a population facing unique health, social, and systemic challenges [20,46]. By systematically mapping the existing literature, this review will identify key findings about care practices, health outcomes, and the barriers faced by both residents and care providers. Importantly, it will reveal significant gaps in knowledge, particularly regarding the integration of HIV care into LTC environments and the psychosocial complexities encountered by this demographic. The insights gained from this review will not only enhance understanding among healthcare providers and policymakers but also guide future research initiatives aimed at optimizing care models for older adults living with HIV. By addressing the identified gaps and emphasizing the need for tailored interventions, this work seeks to promote the provision of high-quality, inclusive care that acknowledges the multifaceted needs of this growing population. Ultimately, the findings from this review have the potential to inform best practices and shape policies that enhance the quality of life for older adults living with HIV in long-term care settings, ensuring that they receive the comprehensive care they deserve.

## Supporting information

S1 File PRISMA-P 2015 Checklist. (DOCX)

S2 File Preferred Reporting Items for Systematic reviews and Meta-Analyses extension for Scoping Reviews (PRISMA-ScR) Checklist. (DOCX)

S3 File PRESS. (DOCX)
