# Supplementary material for: Exploring the state of evidence on aging with HIV in long-term care: A scoping review protocol
Source: PLoS One. 2025 Oct 29;20(10):e0335131. doi: 10.1371/journal.pone.0335131 (PMC12571243; doi:10.1371/journal.pone.0335131)
Supplement: S3 File — (DOCX) [file pone.0335131.s003.docx]

Testing PRESS Suggestions: Scoping Review on HIV & LTC

| **#** | **Suggestion** | **Comments** |
| --- | --- | --- |
|  | HIV Terms |  |
| 1 | AIDS.jw. | Can add (searches the journal name) - in case they don’t mention HIV/AIDS in the title, abstract, keywords |
| 2 | HIV.jw. | Can add (searches the journal name) |
| 3 | (AIDS adj2 encephalit*).tw,kf. | Adds 0 additional articles but will add to search for comprehensiveness |
| 4 | "aids-related".tw,kf. | Already in the search - this was just comment that it may bring up articles that say “hearing aids-related", but I found that this can be easily screened out |
| 5 | "AIDS/HIV".tw,kf. | Not necessary to add since line #9 (hiv.tw,kf) would capture this |
| 6 | "HIV/AIDS".tw,kf. | Same as above |
| 7 | Human T-Cell Leukemia Virus??.tw,kf. | I would not suggest adding this since I already search this but specify that it’s type 3. This line doesn’t add anything in Medline, Added 1 article in Embase but was irrelevant since its HTLV-1 (instead of HTLV-3 which was searched). I think it would be better to not change the search (which specifies it’s type 3) |
| 8 | lymphadenopathy-associated virus*.tw,kf. | Already included in the search |
| 9 | lav-htlv-iii.tw,kf. | Not necessary to add since it’s captured by line #15 (HTLV-III.tw,kf) (adds 0 articles) |
| 10 | ("kaposi?? sarcoma" and "immunoblastic lymphadenopath*").mp. | This doesn’t seem to add anything and is probably too broad - I chose to search “AIDS-related” instead (already int he search) to account for the different diseases that could manifest, e.g. AIDS-related Kaposi sarcoma, AIDS-related dementia |
| 11 | ("cytomegalovirus infection?" or "cytomegalic inclusion disease?").mp. and 1981*:1985.(dt). | I think this would not be necessary to search but because it brings up very few articles, I can add this if you’d like. They are suggesting to put a date limit of 1981-1985 for when this term may be used. When searching this along, there might be articles about cytomegalovirus in homosexual men (which would suggest HIV) but when this line is combined with LTC, I'm only getting articles on kidney transplant or infants. |
| 12 | "pneumocystis carinii pneumonia?".mp. and 1981*:1985.(dt). | I think this would not be necessary to search. They are suggesting to put a date limit of 1981-1985 for when this term may be used. Simialr to above on cytomegalovirus, it doesn’t seem to bring up anything relevant when combined with LTC. |
|  | LTC Terms |  |
| 13 | Geriatric Nursing/ | I’m finding that this MeSH brings up mainly review articles about aging in HIV (which would be excluded because they’re reviews) and for others, the title/abstracts are not clear whether they are about LTC (could be in the home, hospital, or LTC). Since all the suggestions do not increase the yield by a lot, I think this could be added but it would require you to review the full-text to confirm if its about LTC. |
| 14 | Housing for the Elderly/ | I'm hesitant to include this MeSH because the definition appears to be for independent living (<https://www.ncbi.nlm.nih.gov/mesh/?term=housing+for+the+elderly> ) and the MeSH “Homes for the aged” would be more appropriate for LTC and is already included: <https://www.ncbi.nlm.nih.gov/mesh/68006707> ) |
| 15 | Nurses Improving Care for Health System Elders/ | This is a relatively new MeSH; appears to be about a national geriatric nursing program (definition: <https://www.ncbi.nlm.nih.gov/mesh/2027845> ); I don’t think this is relevant (not specific for LTC) and not worth adding (only 2 articles indexed with this so far) |
| 16 | (housing/ or public housing/) and exp aged/ | Similar for housing for the elderly, this is probably too broad; may also include articles about housing status for the elderly. |
| 17 | "home? for the elder*".tw,kf. | Not adding since I have a similar line that is more specific (this suggestion brings up a lot of noise since it includes stop words – i.e. the database will ignore “for” and “the” so it is equivalent to searching home? ____ ____ elderl* where the blanks could mean anyword, while my search ("home? for? the? elder*".tw,kf ) uses wildcards (denoted by ?) to force the database to search “for” and “the”) |
| 18 | (home? adj3 elder*).tw,kf. | This would likely not bring anything relevant but would be broader. Possible that it may bring noise, e.g. “homes occupied by elderly” or “home-based elderly”. The other terms in the search would likely be able to capture everything relevant, e.g. elderly care homes, elderly nursing homes, etc. |
| 19 | "retirement home?".tw,kf. | This adds a few articles => I know I’ve asked this before but I was talking to the reviewer about it and we were thinking that retirement homes could provide different levels of care, e.g. assisted living, depending on the country. |
| 20 | "shelter* housing?".tw,kf. | Sheltered housing seems to be a UK term => Question: is this relevant? |
| 21 | "shelter* home?".tw,kf. | Same as above |
| 22 | (aged adj2 care adj2 facilit*).tw,kf. | Will include to make it more comprehensive, but doesn’t seem to add much |
| 23 | (care adj2 facilit* adj4 aged).tw,kf. | Will include to make it more comprehensive, but doesn’t seem to add much |
| 24 | (institutionali#ed adj2 elder*).tw,kf. | Will include to make it more comprehensive, but doesn’t seem to add much |
| 25 | (residential adj2 service?).tw,kf. | Will include to make it more comprehensive, but doesn’t seem to add much |
| 26 | (residential adj2 (facility or facilities or institution* or home? or centre? or center? or lodge? or **lodging?**)).tw,kf. | Will include to make it more comprehensive, but doesn’t seem to add much  – this line is included but the change is adding the word lodging |
| 27 | (senior? adj (facility or facilities or institution* or home? or centre? or center? or lodge? or **lodging?** or residence? or **residential**)).tw,kf. | Will include to make it more comprehensive, but doesn’t seem to add much  – this line is included but the change is adding the word lodging and residential. |
